# Expression of the G protein-coupled estrogen receptor (GPER) in endometriosis: a tissue microarray study

**DOI:** 10.1186/1477-7827-10-30

**Published:** 2012-04-20

**Authors:** Nicolas Samartzis, Eleftherios P Samartzis, Aurelia Noske, André Fedier, Konstantin J Dedes, Rosmarie Caduff, Daniel Fink, Patrick Imesch

**Affiliations:** 1 Department of Gynecology, University Hospital Zurich, Zurich, Switzerland; 2 Department of Pathology, University Hospital Zurich, Zurich, Switzerland

**Keywords:** GPER, GPR30, Endometriosis, Immunohistochemistry, Tissue microarray

## Abstract

**Background:**

The G protein-coupled estrogen receptor (GPER) is thought to be involved in non-genomic estrogen responses as well as processes such as cell proliferation and migration. In this study, we analyzed GPER expression patterns from endometriosis samples and normal endometrial tissue samples and compared these expression profiles to those of the classical sex hormone receptors.

**Methods:**

A tissue microarray, which included 74 samples from different types of endometriosis (27 ovarian, 19 peritoneal and 28 deep-infiltrating) and 30 samples from normal endometrial tissue, was used to compare the expression levels of the GPER, estrogen receptor (ER)-alpha, ER-beta and progesterone receptor (PR). The immunoreactive score (IRS) was calculated separately for epithelium and stroma as the product of the staining intensity and the percentage of positive cells. The expression levels of the hormonal receptors were dichotomized into low (IRS < 6) and high (IRS > =6) expression groups.

**Results:**

The mean epithelial IRS (+/−standard deviation, range) of cytoplasmic GPER expression was 1.2 (+/−1.7, 0–4) in normal endometrium and 5.1 (+/−3.5, 0–12) in endometriosis (*p* < 0.001), of nuclear GPER 6.4 (+/−2.6, 0–12) and 6.8 (+/−2.9, 2–12; *p* = 0.71), of ER-alpha 10.6 (+/−2.4, 3–12) and 9.8 (+/−3.0, 2–12; *p* = 0.26), of ER-beta 2.4 (+/−2.2; 0–8) and 5.6 (+/−2.6; 0–10; *p* < 0.001), and of PR 11.5 (+/−1.7; 3–12) and 8.1 (+/−4.5; 0–12; *p* < 0.001), respectively. The mean stromal IRS of nuclear GPER expression was 7.7 (+/−3.0; 2–12) in endometrium and 10.8 (+/−1.7; 6–12) in endometriosis (*p* < 0.001), of ER-alpha 8.7 (+/−3.1; 2–12) and 10.6 (+/−2.4; 2–12; *p* = 0.001), of ER-beta 1.8 (+/−2.0; 0–8) and 5.4 (+/−2.5; 0–10; *p* < 0.001), and of PR 11.7 (+/−0.9; 8–12) and 10.9 (+/−2.0; 3–12; *p* = 0.044), respectively. Cytoplasmic GPER expression was not detectable in the stroma of endometrium and endometriosis. The observed frequency of high epithelial cytoplasmic GPER expression levels was 50% (n = 30/60) in the endometriosis and none (0/30) in the normal endometrium samples (*p* < 0.001). High epithelial cytoplasmic GPER expression levels were more frequent in endometriomas (14/20, 70%; *p* = 0.01), as compared to peritoneal (9/18, 50%) or deep-infiltrating endometriotic lesions (7/22, 31.8%). The frequency of high stromal nuclear GPER expression levels was 100% (n = 74/74) in endometriosis and 76.7% (n = 23/30) in normal endometrium (*p* < 0.001). The frequency of high epithelial nuclear GPER expression levels did not differ between endometriosis and normal endometrium.

**Conclusions:**

The present data indicate a unique GPER expression pattern in endometriosis, especially in endometriomas as compared to the normal endometrium. The overexpression of GPER in endometriotic lesions suggests a potential role for GPER in the hormonal regulation of endometriosis, which should be taken into consideration for future hormonal treatment strategies.

## Background

Endometriosis is a common, benign inflammatory disease defined by the presence of endometrial cells outside of the uterine cavity [1]. This disease affects 5 to 10% of women within reproductive age and is clinically characterized by chronic pelvic pain, dysmenorrhea, dyspareunia and infertility [2-4].

Although the exact mechanism of endometriosis pathogenesis has not yet been fully elucidated, estrogens are known to play a key role in this process. Therefore, currently available medical treatment options employ various mechanisms to target the estrogen pathway. The classic biological effects associated with estrogens are mediated by the estrogen receptors (ERs) ER-alpha and ER-beta. Recently, the G protein-coupled estrogen receptor (GPER), which is also known as the G protein-coupled receptor 30 (GPR30), has been described as a novel ER that exhibits a high-affinity binding site for estrogens [5]. GPER is a seven-transmembrane receptor that is thought to be part of the rapid, non-genomic estrogen responses that can, in contrast to the classic or genomic modes of ER activity, occur within minutes [6,7].

The GPER-mediated activity of estrogen is involved in multiple physiological intracellular processes, such as phosphatidylinositol-3-OH kinase activation, calcium mobilization, cyclic AMP production and extracellular signal-dependent kinase activation [8,9]. In cancer cells, GPER may enhance proliferative processes mediated by the actions of estrogens and tamoxifen [7,10].

Bulun et al. demonstrated that the levels of ER-alpha, ER-beta and progesterone-receptor (PR) are markedly different in endometrial cells as compared to endometriosis-derived stromal cells [11]. While the expression levels of ER-beta were observed to be higher in endometriotic stromal cells as compared with endometrial stromal cells, the expression levels of ER-alpha and PR were reported to be higher in endometrial stromal cells. Moreover, GPER expression has not been studied in endometriotic cells to date. As estrogen activity plays a key role in the development of endometriosis, an investigation into GPER expression in endometriotic tissue is warranted. Therefore, the aim of this study was to determine the level of GPER expression in endometriotic cells and to compare this expression level with that of the classical sex receptors ER-alpha, ER-beta and PR.

## Methods

### Patient selection

This study was approved by the local ethics committee (ref. number KEK-ZH-NR 2010-0174/0). Patients were retrospectively included in this study after receiving a biopsy-proven diagnosis of endometriosis. Additional inclusion criteria included the availability of adequate tissue quantity, exclusively proliferative cell cycle status and a lack of supplemental hormonal intake (neither GnRH analogs nor oral contraceptives). All specimens were paraffin-embedded in tissue blocks, and the histological diagnosis of endometriosis was conducted between 2000 and 2010 at the Institute of Pathology of the University Hospital in Zurich. These diagnoses were reviewed by a gynecological pathologist (R.C.). In accordance with the inclusion criteria of the study, 71 patients with endometriosis and 30 control patients with normal endometria and no diagnosis of endometriosis were identified. The control cases included patients who had undergone hysterectomies for benign conditions other than diseases affecting the endometrium or endometriosis. A total of 74 endometriotic specimens (three patients each provided two separate samples of endometriotic tissue from distinct localizations) and 30 normal endometrial specimens were examined in the study. Histological specimens were obtained from patients with ovarian endometriomas (n = 27), peritoneal endometriosis (n = 19) and deep-infiltrating endometriosis (n = 28).

### Tissue microarray construction

A tissue microarray (TMA) was constructed using a semiautomatic tissue arrayer (Beecher Instruments, Woodland, USA), as previously described [12,13]. Tissue areas exhibiting endometriosis were marked on hematoxylin/eosin-stained sections. Cylindrical cores of 0.6 mm in diameter were punched out of the corresponding paraffin-embedded blocks and inserted into a recipient block. Two different spots from each patient sample were punched out.

### Immunohistochemistry

TMA sections (2.5-μm thick) were transferred to glass slides for the immunohistochemical (IHC) analysis, according to the Ventana automated protocols. The following antibodies were used for IHC analysis: monoclonal rabbit antibodies against ER-alpha (prediluted and obtained from Ventana Medical System Inc., clone SP1), monoclonal mouse antibodies against ER-beta (diluted at 1:150 and obtained from Gene Tex, Inc., clone 14 C8), monoclonal mouse antibodies against PR (prediluted and obtained from Ventana Medical System, Inc., clone 1A6) and polyclonal rabbit antibodies against GPER (diluted at 1:50 and obtained from Abcam Limited, code ab39742). Normal breast tissue and breast cancer tissue served as positive controls for GPER expression. Normal endometrial tissue was used as a positive control for ER and PR expression. Negative controls were performed by omitting the primary antibody.

### Scoring

The TMA was scored independently by two observers (A.N. and N.S.) who were blinded to the clinicopathological information associated with the samples. Two tissue cores from each individual case were evaluated. The expression of ER (alpha and beta), PR, and GPER was evaluated separately for epithelium and stroma according to the percentage of positive cells and the intensity of the staining. The percentage of positive cells was scored using the following numeric system: 0 (0% positive cells); 1 (<10%); 2 (11-50%); 3 (51-80%); and 4 (>80%). The staining intensity was scored as either 0 (negative), 1 (weak), 2 (moderate) or 3 (strong). For the immunoreactive score (IRS) [14], the percentage of positive cells and the staining intensity were multiplied, which produced a value for the IRS between 0 and 12. To separate cases with a weak or strong immunoreaction, we used a median nuclear GPER IRS of 6 as the cutoff point for dichotomization. The same cutoff value was used for the other receptors, and this created a “high expression” group (IRS of 6 or more) and a “low expression” group (IRS less than 6) [15].

### Statistical analysis

The results are presented as the absolute numbers and percentages and as the mean values, standard deviations and ranges. P-values ≤0.05 were considered statistically significant if not indicated differently. SPSS (version 19, SPSS Inc., Chicago, IL) was utilized for the statistical analysis. Non-parametric tests were used to compare the IRS values between the different sample groups. Therefore, a Wilcoxon rank-sum test was used to compare the IRS in endometrium and endometriosis. The significance levels for the endometrium and the subgroups of endometriosis were assessed by a Kruskal-Wallis one-way analysis of variance followed post-hoc by a pairwise Wilcoxon rank-sum test. In these cases, a Bonferroni correction (α’ = α/6) was performed and p-values were only considered significant if *p* ≤ 0.0083. Frequencies of low and high expression levels were analyzed by an exact 2-sided Pearson chi-square test. Bivariate correlation analysis was done with Spearman’s rho.

## Results

### Patients’ clinical characteristics

The mean age (±standard deviation, range) of the patients in the endometriosis cohort was 33.9 (±5.8, 19–48) years, as compared to 39.5 (±3.3, 29–44) years for patients in the control group. Regarding the different endometriosis subtypes the mean age was 35.2 (±6.9, 19–48) years for patients with ovarian endometriosis, 31.3 (±4.0, 25–38) years for patients with peritoneal endometriosis, and 34.5 (±5.4, 25–42) years for patients with deep-infiltrating endometriosis. Endometriosis staging, according to the classifications of the American Society for Reproductive Medicine [16], had been assigned as follows: stage I for 5/74 cases (6.8%); stage II for 7/74 cases (9.5%); stage III for 15/74 cases (20.3%); stage IV for 34/74 cases (45.9%); and N/A for 13/74 (17.5%) cases. The patients’ body mass indexes (BMIs) were available in 51/104 (49%) of the cases, and these exhibited mean values of 22.0 (±5.0, 16–40) kg/m^2^ for patients in the endometrioma group, 24.2 (±7.5, 20–45) kg/m^2^ for the peritoneal endometriosis group, 25.1 (±10.0, 19–47) kg/m^2^ for the deep-infiltrating endometriosis group and 23.5 (±3.7, 16–30) kg/m^2^ for the control group.

### Immunohistochemical analysis

**Figure 1:**
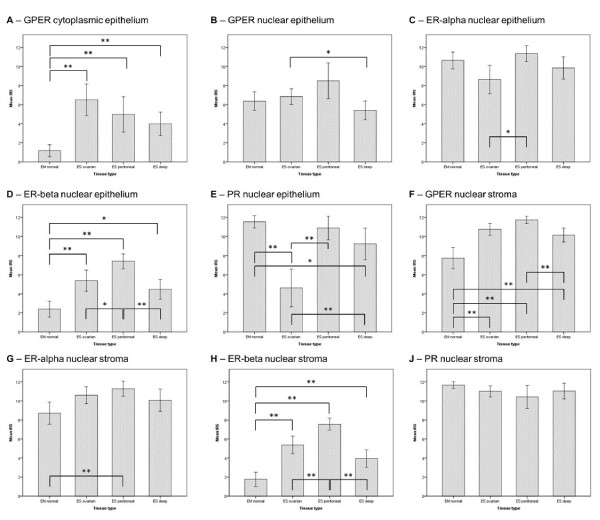
**Immunoreactivity Scores.** Mean Immunoreactivity Scores (IRS) and 95% confidence intervals (error bars) for the expression of the (**A**) cytoplasmic expression of the G protein-coupled estrogen receptor (GPER cytoplasmic), the (**B**) nuclear expression of the G protein-coupled estrogen receptor (GPER nuclear), the (**C**) estrogen receptor alpha (ER-alpha), the (**D**) estrogen receptor beta (ER-beta), and the (**E**) progesterone receptor (PR) in the epithelium of normal endometrium (normal EM), ovarian endometriosis (ovarian ES), peritoneal endometriosis (peritoneal ES) and deep-infiltrating endometriosis (deep ES), as well as (**F**-**J**) the same receptors in the stroma of the mentioned tissues. Stromal cells did not show cytoplasmic GPER expression in endometrium and endometriosis and hence no corresponding chart is represented in this figure. Significance (*) was considered if *p* ≤ 0.0083 in the pairwise analysis according to a Bonferroni correction (α’ = α/6). Highly significant values (*p* ≤ 0.0016) are marked with (**).

**Figure 2:**
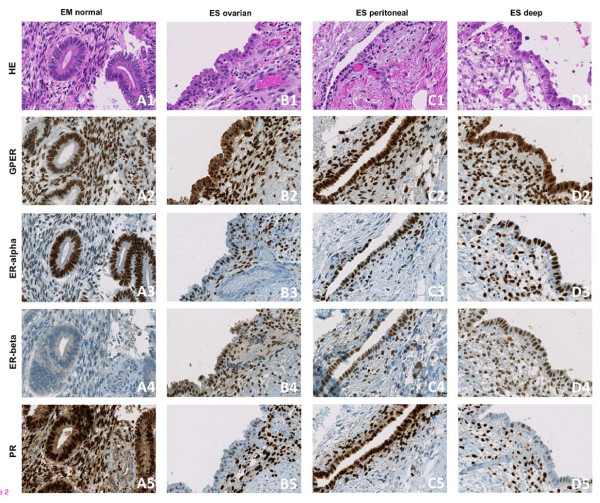
**Representative histological tissue samples.** Representative tissue samples of a normal endometrium (EM normal) microarray core (A1 – A5), an ovarian endometriosis (ES ovarian) tissue core (B1 – B5), a peritoneal endometriosis (ES peritoneal) tissue core (C1 – C5) and a deep-infiltrating endometriosis (ES deep) tissue core (D1 – D5). The samples were stained with hematoxylin/eosin (HE) (A1, B1, C1, D1), as well as by immunohistochemistry to detect the G protein-coupled estrogen receptor (GPER) (A2, B2, C2, D2), the estrogen receptor alpha (ER-alpha) (A3, B3, C3, D3), the estrogen receptor beta (ER-beta) (A4, B4, C4, D4) and the progesterone receptor (PR) (A5, B5, C5, D5) expression; Magnification × 200.

**Figure 3:**
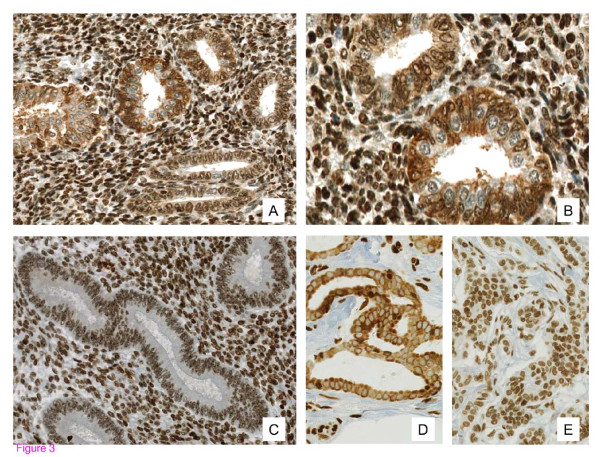
**Expression of the G protein-coupled estrogen receptor (GPER) in endometriosis, in normal endometrium and in the positive control. (A-C)** Expression of the G protein-coupled estrogen receptor (GPER) in ovarian endometriosis with strong cytoplasmic and partial nuclear expression in the epithelium and strong nuclear expression in the stroma (**A**, 200×; **B**, 400×), compared to normal endometrium with positive nuclear and lack of cytoplasmic GPER expression (**C**, 200×). **(D-E)** GPER expression from breast carcinoma as a positive control with (**D**, 200×) strong cytoplasmic and negative nuclear GPER expression, as well as with (**E**, 200×) weak cytoplasmic and strong nuclear GPER expression.

Immunohistochemical (IHC) staining of GPER, ER-alpha, ER-beta, and PR was evaluable in 86.5% (n = 90/104), 89.4% (93/104), 91.3% (95/104), and 86.5% (90/104) of all cases for the epithelial parts and in 100% (n = 104/104), 96.2% (n = 100/104), 97.1% (n = 101/104), and 98.1% (n = 102/104) for the stromal parts, respectively. The remaining cases were not evaluable mainly due to a lack of adequate tissue (either epithelial, stromal, or both) from the respective samples. We observed (in contrast to findings regarding ER/PR expression) nuclear and cytoplasmic expression of GPER in the epithelium of endometriotic lesions. As a result, both of these staining patterns were analyzed separately. Membrane-specific staining of GPER was not detectable in the endometrial or the endometriotic tissue samples. The mean epithelial IRS (+/−standard deviation, range) of cytoplasmic GPER expression was 1.2 (+/−1.7, 0–4) in normal endometrium and higher with 5.1 (+/−3.5, 0–12) in endometriosis (*p* < 0.001). The mean epithelial IRS of nuclear GPER expression was comparable with 6.4 (+/−2.6, 0–12) in the endometrium and 6.8 (+/−2.9, 2–12) in endometriosis (*p* = 0.71). ER-alpha showed a mean epithelial IRS of 10.6 (+/−2.4, 3–12) in the endometrium and of 9.8 (+/−3.0, 2–12) in endometriosis (*p* = 0.26). The mean epithelial IRS of ER-beta was 2.4 (+/−2.2; 0–8) in endometrium and higher (*p* < 0.001) in endometriosis with 5.6 (+/−2.6; 0–10). The mean epithelial IRS of PR was higher (*p* < 0.001) in the endometrium with 11.5 (+/−1.7; 3–12) than in endometriosis 8.1 (+/−4.5; 0–12). The mean stromal IRS of nuclear GPER expression was 7.7 (+/−3.0; 2–12) in normal endometrium and 10.8 (+/−1.7; 6–12) in endometriosis (*p* < 0.001). Cytoplasmic GPER expression was not detectable in the stroma of endometrium and endometriosis. The mean stromal IRS of ER-alpha was 8.7 (+/−3.1; 2–12) in normal endometrium and 10.6 (+/−2.4; 2–12) in endometriosis (*p* = 0.001). The mean stromal IRS of ER-beta was 1.8 (+/−2.0; 0–8) in endometrium and higher (*p* < 0.001) in endometriosis with 5.4 (+/−2.5; 0–10) for ER-beta. The mean stromal IRS of PR was 11.7 (+/−0.9; 8–12) in normal endometrium and 10.9 (+/−2.0; 3–12) in endometriosis (*p* = 0.044). The mean IRS values for all subtypes of endometriosis are reported in Table 1 and represented as charts in Figure 1. Representative histological images are depicted in Figure 2 and more detailed histological sections for GPER are shown in Figure 3 including positive control tissue samples.

**Table 1:** Mean immunoreactivity scores of the expression of GPER, ER alpha, ER beta and PR in the epithelium and stroma of normal endometrium and different endometriosis types

	**Normal Endometrium**	**Ovarian Endometriosis**	**Peritoneal Endometriosis**	**Deep-Infiltrating Endometriosis**
**Epithelium**				
GPER cyt	1.2 (±1.7, 0–4)	6.5 (±3.5, 0–12)	5.0 (±3.7, 0–10)	4.0 (±2.8, 0–8)
GPER nuc	6.4 (±2.6, 0–12)	6.9 (±1.8, 3–8)	8.5 (±3.8, 2–12)	5.4 (±2.2, 2–12)
ER alpha	10.6 (±2.4, 3–12)	8.6 (±3.5, 2–12)	11.4 (±1.6, 6–12)	9.8 (±2.7, 3–12)
ER beta	2.4 (±2.2, 0–8)	5.4 (±2.6, 0–10)	7.4 (±1.5, 4–10)	4.5 (±2.5, 0–8)
PR	11.5 (±1.7, 3–12)	4.6 (±4.3, 0–12)	10.9 (±2.4, 3–12)	9.2 (±3.7, 2–12)
**Stroma**				
GPER nuc	7.7 (±3.0, 2–12)	10.7 (±1.6, 8–12)	11.7 (±0.8, 9–12)	10.1 (±1.9, 6–12)
ER alpha	8.7 (±3.1, 2–12)	10.6 (±2.1, 6–12)	11.3 (±1.6, 6–12)	10.1 (±3.0, 2–12)
ER beta	1.8 (±2.0, 0–8)	5.4 (±2.3, 0–10)	7.6 (±1.3, 4–10)	3.9 (±2.3, 0–8)
PR	11.7 (±0.9, 8–12)	11 (±1.4, 8–12)	10.4 (±2.5, 3–12)	11 (±2.1, 4–12)

Values are represented as mean immunoreactivity scores (IRS) (±standard deviation, range). *GPER* G protein-coupled estrogen receptor (*cyt* cytoplasmic, *nuc* nuclear staining), *ER* estrogen receptor, *PR* progesterone receptor. No cytoplasmic GPER expression was detected in the stroma of endometrium and endometriosis.

### Frequencies of low and high expression groups

Following dichotomization in low and high expression groups, we analyzed the frequency of the cases. High epithelial expression of cytoplasmic GPER was significantly more frequent (*p* < 0.001) in endometriotic tissues (n = 30/60, 50%) than in eutopic endometrial tissues (n = 0/30). In regards of the different endometriosis subtypes, high cytoplasmic GPER expression levels were more frequent in endometriomas (14/20, 70%; *p* = 0.01), as compared to peritoneal (9/18, 50%) or deep-infiltrating endometriotic lesions (7/22, 31.8%). However, no significant differences between endometriotic and eutopic endometrial samples were observed in regards to the nuclear expression of GPER in the epithelium. In the stroma the frequency of high nuclear GPER expression levels was 100% (n = 74/74) in endometriosis and 76.7% (n = 23/30) in normal endometrium (*p* < 0.001). High ER-beta expression levels were more frequent in endometriosis than in eutopic endometrial cells (*p* < 0.001) for both, stroma and epithelium. Low PR expression levels were seen more frequently in the epithelium of endometriosis (*p* = 0.005) but not in the stroma (*p* = 0.6). The differences in the epithelial PR expression levels were mainly associated with the lower expression ovarian endometriosis, as shown in Figure 1. More cases of low epithelial expression levels of ER-alpha and PR (21.7% and 61.9%, respectively) were observed in samples of ovarian endometriosis, as compared with those of peritoneal endometriosis (0% and 5.9%, respectively) and deep-infiltrating endometriosis (4.3% and 18.2%, respectively; p-value of 0.02 and <0.001). All expression level frequencies for cytoplasmic and nuclear GPER, ER-alpha, ER-beta and PR in epithelium and stroma of endometrium and endometriosis including the endometriosis subtypes are reported in detail in Tables 2, 3, 4 and 5.

**Table 2:** Frequencies of the epithelial expression levels of GPER (nuclear and cytoplasmic) and the classical sex hormone receptors in normal endometrium and endometriosis

**Receptor**	**Expression level**	**Endometrium**	**Endometriosis**	**p-value**
GPER cyt	High	0/30	30/60 (50%)	**<0.001**
	Low	30/30 (100%)	30/60 (50%)	
GPER nuc	High	19/30 (63.3%)	42/60 (70%)	0.6
	Low	11/30 (36.7%)	18/60 (30%)	
ER-alpha	High	28/30 (93.3%)	57/63 (90.5%)	0.7
	Low	2/30 (6.7%)	6/63 (9.5%)	
ER-beta	High	3/30 (10%)	35/65 (53.8%)	**<0.001**
	Low	27/30 (90%)	30/65 (46.2%)	
PR	High	29/30 (96.7%)	42/60 (70%)	**0.005**
	Low	1/30 (3.3%)	18/60 (30%)	

*GPER* G protein-coupled estrogen receptor (*cyt* cytoplasmic, *nuc* nuclear staining), *ER* estrogen receptor, *PR* progesterone receptor. P-Values were calculated by an exact 2-sided Pearson chi-square test; *p* ≤ 0.05 was considered as statistically significant and is reported in bold type.

**Table 3:** Frequencies of the stromal expression levels of GPER (nuclear and cytoplasmic) and the classical sex hormone receptors in normal endometrium and endometriosis

**Receptor**	**Expression level**	**Endometrium**	**Endometriosis**	***p*-value**
GPER nuc	High	23/30 (76.7%)	74/74 (100%)	**<0.001**
	Low	7/30 (23.3%)	0/74	
ER-alpha	High	25/30 (83.3%)	68/70 (97.1%)	**0.02**
	Low	5/30 (16.7%)	2/70 (2.9%)	
ER-beta	High	2/30 (6.7%)	35/71 (49.3%)	**<0.001**
	Low	28/30 (93.3%)	36/71 (50.7%)	
PR	High	30/30 (100%)	70/72 (97.2%)	0.6
	Low	0/30	2/72 (2.8%)	

*GPER* G protein-coupled estrogen receptor (*nuc* nuclear staining); *ER* estrogen receptor; *PR* progesterone receptor. No cytoplasmic GPER expression was detected in the stroma of endometrium and endometriosis. P-Values were calculated by an exact 2-sided Pearson chi-square test; *p* ≤ 0.05 was considered as statistically significant and is reported in bold type.

**Table 4:** Frequencies of the epithelial expression levels of GPER (nuclear and cytoplasmic) and the classical sex hormone receptors in different types of endometriosis

**Receptor**	**Expression level**	**Ovarian Endometriosis**	**Peritoneal Endometriosis**	**Deep infiltrating Endometriosis**	**p-value**
GPER cyt	High	14/20 (70.0%)	9/18 (50.0%)	7/22 (31.8%)	**0.01**
	Low	6/20 (30.0%)	9/18 (50.0%)	15/22 (68.2%)	
GPER nuc	High	16/20 (80.0%)	13/18 (72.2%)	13/22 (59.1%)	0.2
	Low	4/20 (20.0%)	5/18 (27.8%)	9/22 (40.9%)	
ER-alpha	High	18/23 (78.3%)	17/17 (100%)	22/23 (95.7%)	**0.02**
	Low	5/23 (21.7%)	0/17	1/23 (4.3%)	
ER-beta	High	11/24 (45.8%)	15/17 (88.2%)	9/24 (37.5%)	**0.001**
	Low	13/24 (54.2%)	2/17 (11.8%)	15/24 (62.5%)	
PR	High	8/21 (38.1%)	16/17 (94.1%)	18/22 (81.8%)	**<0.001**
	Low	13/21 (61.9%)	1/17 (5.9%)	4/22 (18.2%)	

*GPER* G protein-coupled estrogen receptor (*cyt* cytoplasmic, *nuc* nuclear staining), *ER* estrogen receptor; *PR* progesterone receptor. P-Values were calculated by an exact 2-sided Pearson chi-square test; *p* ≤ 0.05 was considered as statistically significant and is reported in bold type.

**Table 5:** Frequencies of the stromal expression levels of GPER (nuclear and cytoplasmic) and the classical sex hormone receptors in different types of endometriosis

**Receptor**	**Expression level**	**Ovarian Endometriosis**	**Peritoneal Endometriosis**	**Deep infiltrating Endometriosis**	**p-value**
GPER nuc	High	27/27 (100%)	19/19 (100%)	28/28 (100%)	-
	Low	0/27	0/19	0/28	
ER-alpha	High	25/25 (100%)	18/18 (100%)	25/27 (92.6%)	0.2
	Low	0/25	0/18	2/27 (7.4%)	
ER-beta	High	11/27 (40.7%)	17/18 (94.4%)	7/26 (26.9%)	**<0.001**
	Low	16/27 (59.3%)	1/18 (5.6%)	19/26 (73.1%)	
PR	High	26/26 (100%)	18/19 (94.7%)	26/27 (96.3%)	0.3
	Low	0/26	1/19 (5.3%)	1/27 (3.7%)	

*GPER*, G protein-coupled estrogen receptor (*nuc* nuclear staining); *ER* estrogen receptor; *PR* progesterone receptor. No cytoplasmic GPER expression was detected in the stroma of endometrium and endometriosis. P-Values were calculated by an exact 2-sided Pearson chi-square test; *p* ≤ 0.05 was considered as statistically significant and is reported in bold type.

### Correlations of the GPER expression with the classic sex-hormone receptors

The relationship between GPER expression and the expression of ER-alpha, ER-beta and PR was evaluated using Spearman’s correlation coefficients (CC). Significant, positive correlations were observed between nuclear and cytoplasmic GPER expression and nuclear ER-beta expression in epithelial cells (CC = 0.221 and 0.569, p-values of 0.037 and <0.001, respectively), as well as between the stromal expression of GPER and the stromal expression of ER-beta (CC = 0.703, *p* < 0.001). Cytoplasmic GPER expression levels in epithelial cells negatively correlated with epithelial PR expression levels (CC = −0.290, *p* = 0.006), and stromal nuclear GPER expression levels negatively correlated with stromal ER-alpha expression levels (CC = −0.511, *p* < 0.001). The ER-alpha expression levels correlated with the PR expression levels in both the epithelial and stromal compartments, as demonstrated by CC values of 0.448 and 0.259 (p-values <0.001 and 0.009), respectively.

## Discussion

Endometriosis is considered an estrogen-dependent disease, and most current medical therapeutic options target aspects of estrogen stimulus [1-4]. The classic estrogen signaling pathway is thought to be mediated by two nuclear estrogen receptors (ER-alpha and ER-beta), which are encoded by different genes [11,17]. These ligand-activated receptors act as transcription factors to mediate a number of estrogen-dependent responses, and the relative abundance of these two receptors differs between eutopic and ectopic endometrial tissues.

In the present study, we observed different expression levels for the established ERs and the PR in endometriotic tissue; there was a relative upregulation in the expression of ER-beta in endometriotic epithelium and stroma, as well as a downregulation in the epithelial expression of PR. This result is in line with several studies that have reported elevated levels of ER-beta and postulated a functional progesterone resistance in endometriotic tissues in ectopic endometrial tissue [11].

In addition to its relatively slow genomic mechanism of action, estrogen can also act via a more rapid, non-genomic mechanism. In recent years, the GPER has been shown to mediate estrogen signaling in various cell types and is involved in the non-genomic estrogen signaling pathway [18].

In the present study, we demonstrated that GPER expression is detectable in the cytoplasm and the nucleus of endometrial and endometriotic cells. Epithelial and stromal endometrial and endometriotic cells exhibited a distinct expression pattern of GPER. Cytoplasmic GPER expression was significantly higher in the epithelium of all types of endometriosis (peritoneal, ovarian and deep infiltrating endometriotic tissues) compared to normal endometrium. Interestingly, we observed an equal distribution of GPER in the nuclei of epithelial eutopic and ectopic endometrial cells, including the various endometriotic tissue types examined. In contrast to the epithelium, cytoplasmic GPER expression was not detectable in stromal endometrial and endometriotic cells. Nuclear cytoplasmic GPER expression was significantly increased in the stroma of endometriosis compared to normal endometrial stroma.

To date, the localization of GPER has been a subject of debate. Some authors have argued that GPER is localized to the plasma membrane, while others have suggested that the receptor is located within the endoplasmic reticulum, as indicated by microscopy studies using fluorescence-labeled 17-alpha-substituted estrogen derivatives [8,19-22]. Additionally, in a recent study, the nuclear localization of GPER was demonstrated in fibroblasts, indicating that GPER also mediates a nuclear signaling pathway [23]. Moreover, two recent studies demonstrated nuclear GPER expression in eutopic endometrial cells [24,25].

One limitation of the current study was the sole evaluation of the level of functional protein during the proliferative phase of the menstrual cycle. Further evaluations are required to clarify whether there are different expression levels of mRNA and functional protein during the menstrual cycle.

Dennis et al. observed that the GPER-specific antagonist G15 was capable of partially inhibiting the estrogen-dependent proliferation of uterine epithelia [26] and concluded that GPER partially mediates the estrogen-induced proliferative response of the uterine epithelium. In this context, the upregulation of GPER expression in endometriotic lesions could play a crucial role in the pathogenesis of endometriosis, especially considering that ligand stimulation of GPER can activate multiple signaling pathways, including the adenyl cyclase, Src and sphingosine kinase pathways [5,27]. Recent studies have demonstrated the transactivation of the epidermal growth factor receptor (EGFR) following activation by GPER [28], and EGFR activation can control multiple downstream events, such as the activation of the mitogen-activated protein kinases (MAPKs) and phosphatidylinositol 3-kinases (PI3Ks). MAPKs and PI3Ks are involved in numerous cytosolic pathways that, when activated, often lead to cell proliferation, migration and adhesion, which are important steps in the pathogenesis of endometriosis [27]. Knowledge about the expression of GPER is therefore of special interest for better understanding of regulative mechanisms in the pathogenesis of endometriosis.

## Conclusions

The aim of the present study was to identify the natural distribution and expression pattern of the novel estrogen receptor GPER among eutopic endometrial cells and endometriotic cells. The finding that GPER expression was upregulated in endometriotic cells as compared to normal endometrial cells may be important for understanding the pathogenesis of endometriosis. The differences in the epithelial GPER expression levels frequency within the various types of endometriosis, i.e., the more frequent high cytoplasmic expression levels in epithelial ovarian endometriotic cells, as well as the differences in epithelial and stromal expression patterns, appear to contradict the classification of endometriosis as a homogenous disease. Finally, the detection of specific GPER receptor antagonists may provide novel therapeutic treatment options for endometriosis.

## Misc

Nicolas Samartzis and Eleftherios P Samartzis are contributed equally to this study

## Competing interests

The authors declare that they have no competing interests.

## Authors’ contributions

NS and EPS constructed the tissue microarray (TMA), scored the immunohistochemical (IHC) staining, performed statistical analyses and drafted the manuscript. AN and RC revised diagnosis of the paraffin embedded tissue samples and analyzed the IHC-staining of the TMA. AF contributed to the interpretation of data. KJD and DF assisted with the interpretation of data and contributed to the draft of the manuscript. PI conceived the study, collected data and coordinated the procedures during this study. All authors read and approved the final manuscript.
